# Multifocal Head and Neck Tumor in a Patient With HIV: A Rare Case

**DOI:** 10.7759/cureus.75367

**Published:** 2024-12-09

**Authors:** Aminath Afaa Mohamed, Fara Rahidah Hussin, Ummu Afeera Zainulabid, Isa Mohamed Rose, Petrick Ramesh Periyasamy

**Affiliations:** 1 Internal Medicine, National University of Malaysia, Kuala Lumpur, MYS; 2 Internal Medicine, Tuanku Mizan Armed Forces Hospital, Kuala Lumpur, MYS; 3 Pathology and Laboratory Medicine, National University of Malaysia, Kuala Lumpur, MYS

**Keywords:** art, ebv, hiv, immunocompromised, smooth muscle tumor

## Abstract

Epstein-Barr virus-associated smooth muscle tumors (EBV-SMTs) are a rare type of tumor occurring exclusively in immunocompromised patients in the setting of HIV/AIDS, post-organ transplant, and congenital immunodeficiency. These tumors require demonstration of EBV DNA on histopathologic studies in order to establish a diagnosis. The overall prognosis is good. The principle of treatment is re-establishing the host immunity, which includes antiretroviral therapy (ART) in HIV/AIDS patients and reducing immunosuppressive therapy in post-transplant patients. The role of surgery is well established when the tumor is causing a mass effect, whereas chemotherapy and radiotherapy have a limited role. Herein, we report a case of a multifocal EBV-SMT in a patient with HIV, treated successfully with standard ART along with diagnostic and therapeutic surgical resection.

## Introduction

Epstein-Barr virus-associated smooth muscle tumors (EBV-SMTs) are a rare type of tumor occurring in immunocompromised patients, almost exclusively reported in human immunodeficiency virus (HIV)-infected patients, post-organ transplant patients, and patients with congenital immunodeficiency syndromes [1]. The estimated prevalence of this extremely rare tumor is <2 cases per million [2]. It was recognized in 1995 that these tumors were first linked to EBV infection, by demonstrating EBV DNA in tumor tissue using in situ hybridization. The EBV receptor (CD21) has been identified as a prerequisite for EBV infection of smooth muscle cells. It was suggested that cell fusion with EBV-infected lymphocytes was the route of viral entry into non-lymphoid cells [3,4]. These tumors have a predilection to be multifocal and occur in unusual sites as compared to conventional SMTs [5,6]. We report a case of an EBV-SMT in a patient with HIV presenting with multifocal lesions in the brain in addition to a tonsillar mass.

## Case presentation

**Figure 1:**
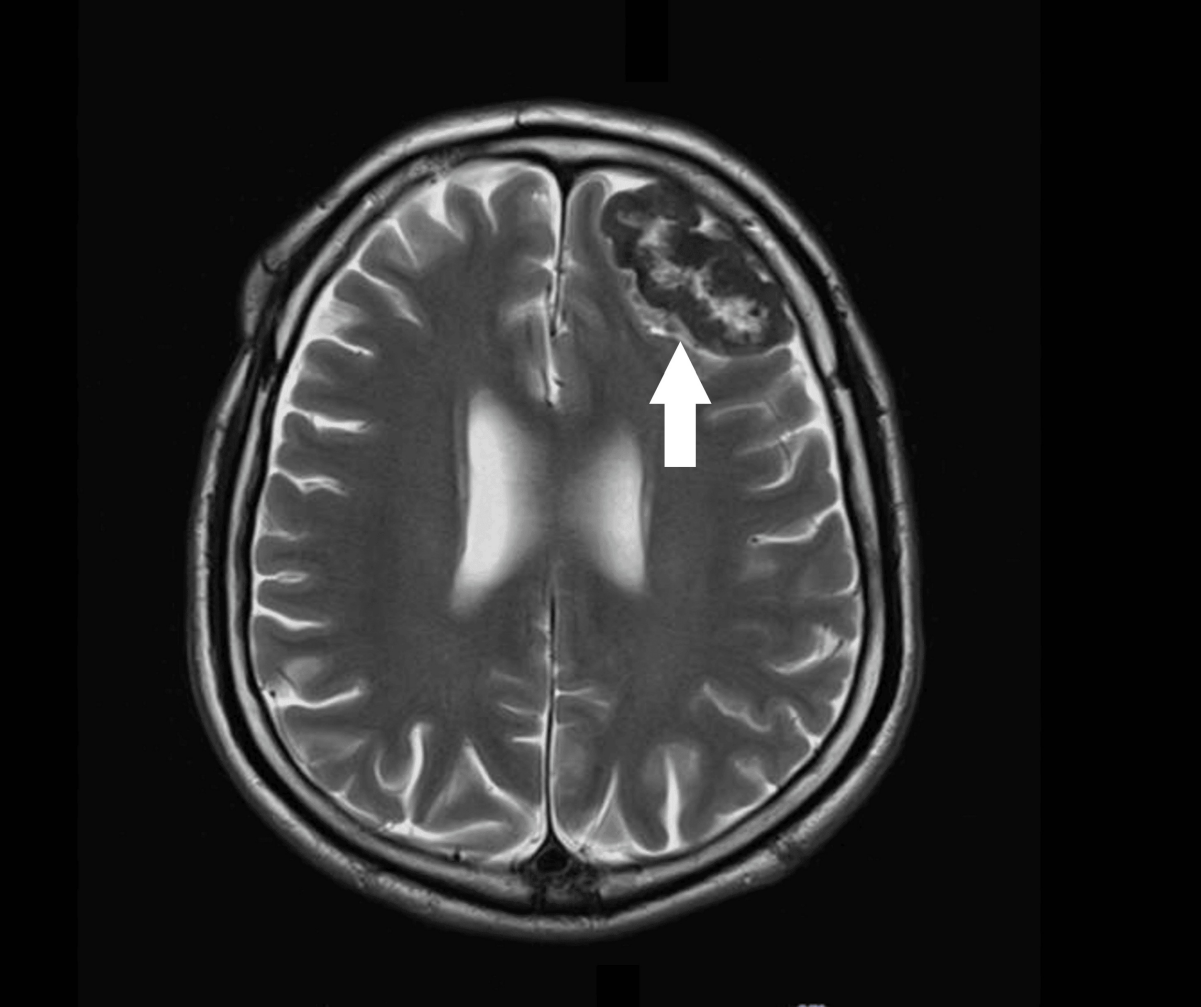
MRI brain with contrast: Axial-view T2-weighted image showing a left frontal lobe lesion

**Figure 2:**
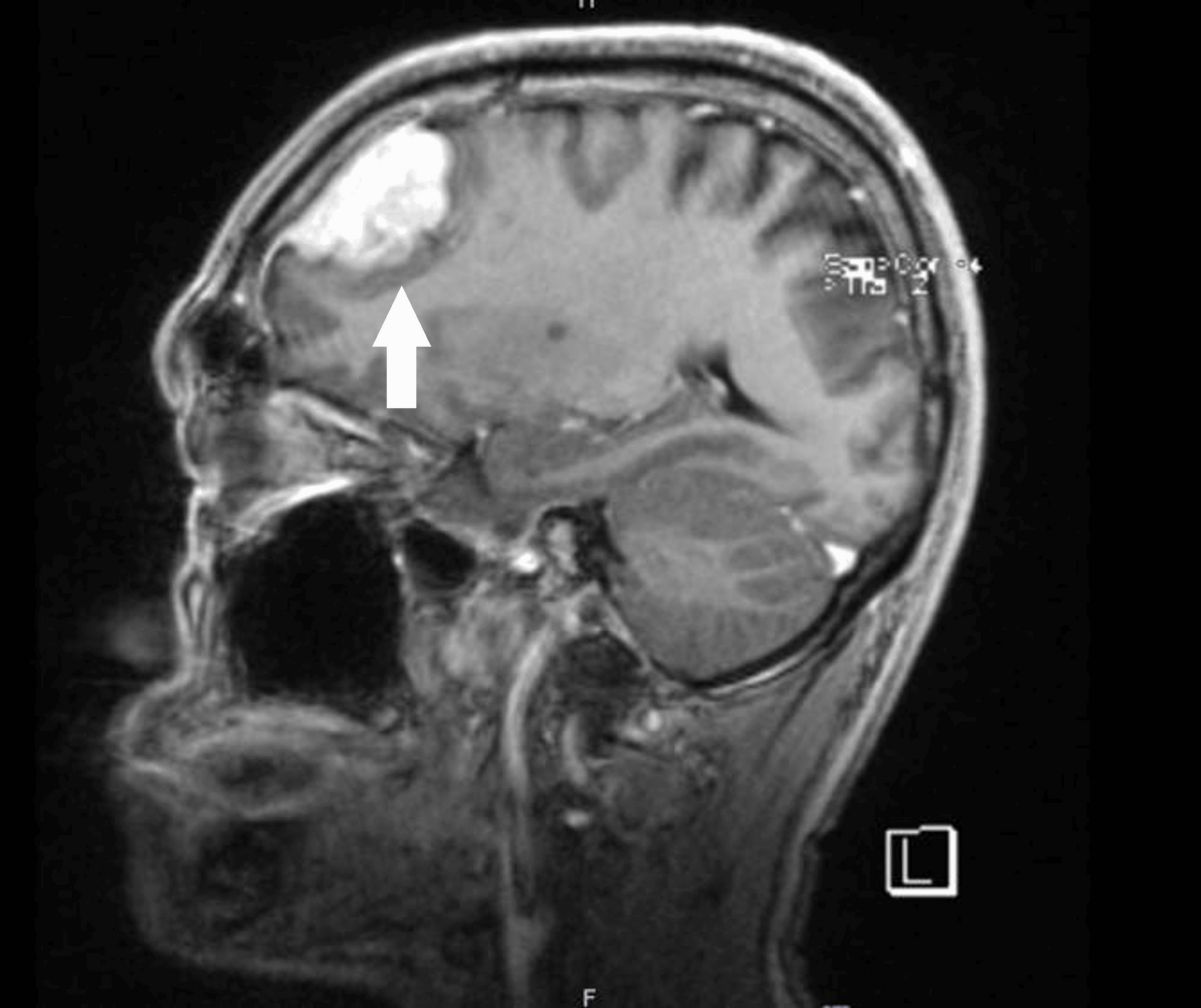
Sagittal-view T1-weighted image showing a left frontal lobe lesion

**Figure 3:**
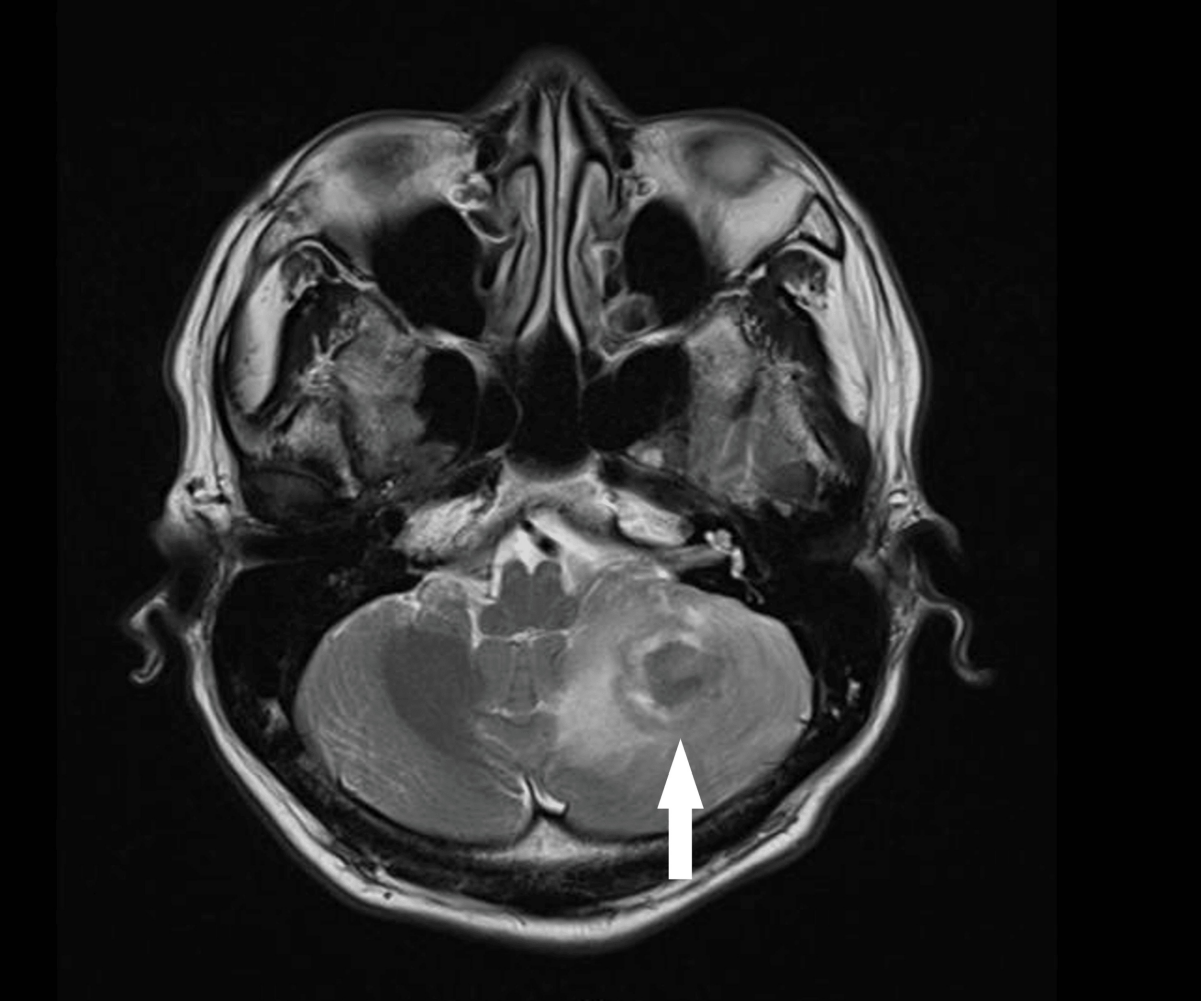
Axial-view T1-weighted image showing a left cerebellar lesion

**Figure 4:**
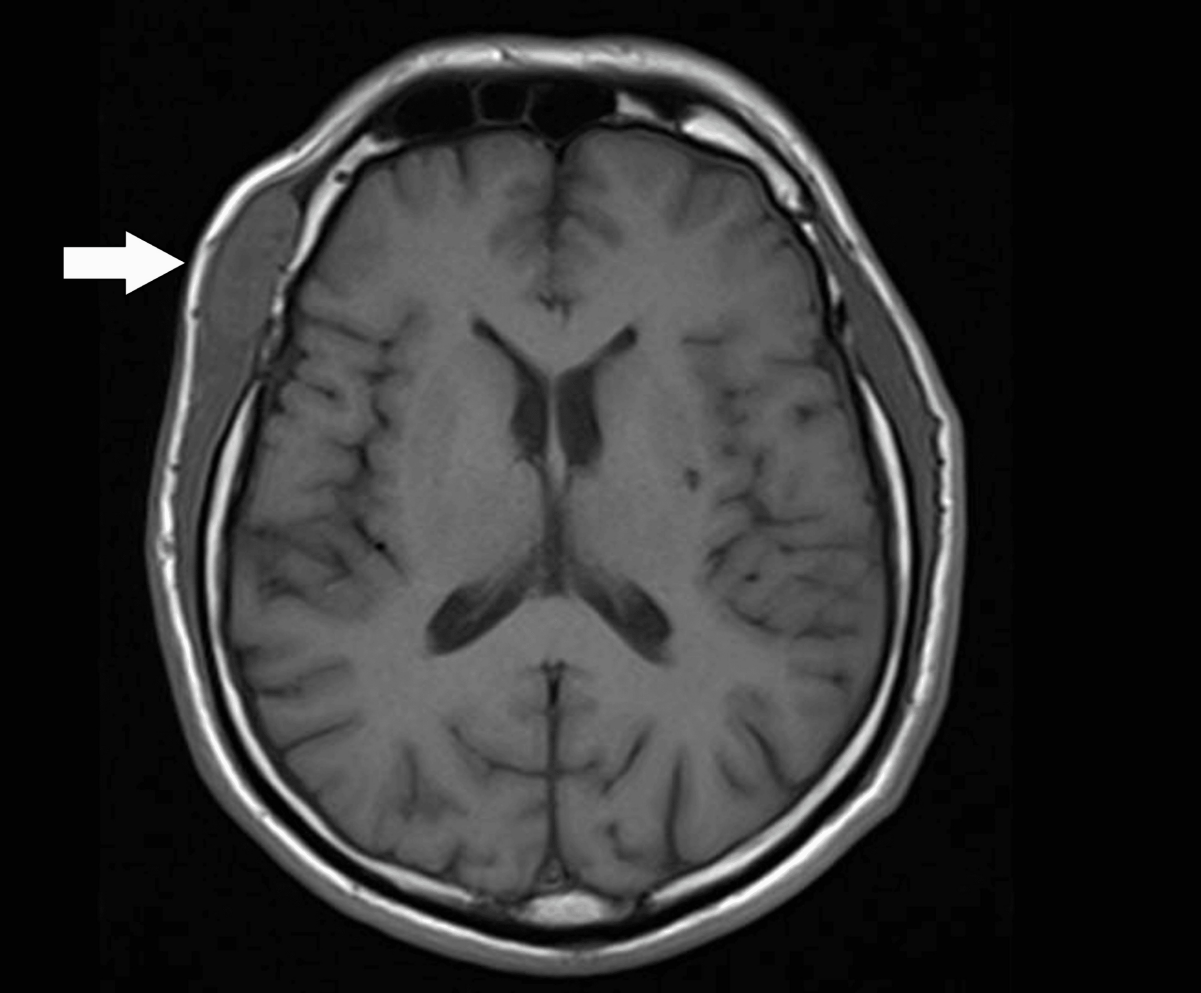
Axial-view T1-weighted image showing a right temporalis muscle lesion

A 51-year-old male patient with underlying retroviral disease was diagnosed in 2015 and started on antiretroviral therapy (ART) with lamivudine-zidovudine-efavirenz. However, he defaulted to follow-up for four years since 2018. At this current admission, he presented with a one-week history of nausea, vertigo, and unsteady gait. Upon arrival to the emergency department, clinical examination revealed a right tonsillar mass, left cranial nerve palsy, nystagmus to the left side, and dysmetria on the left finger-nose test. His routine blood investigations were within normal limits. His serum toxoplasma IgG antibody was positive. His CD4 T lymphocytes or helper T cells were 74 cells/uL and his HIV RNA PCR viral load of 146000 copies/ml. Based on his latest HIV RNA PCR viral load, he possibly developed virological failure which means previous ART failed to suppress and sustain viral load to less than 200 copies/ml. Lumbar puncture also was done and cerebrospinal fluid (CSF) examination revealed a high protein of 1816 mg/L with normal glucose, negative bacterial, tuberculosis (TB), and fungal cultures, benign cytology and positive DNA PCR for CSF EBV. Magnetic resonance imaging (MRI) of the brain revealed multiple extracranial and intracranial lesions involving the right temporalis muscle, left frontal lobe, and left cerebellum (Figures 1-4). 

**Figure 5:**
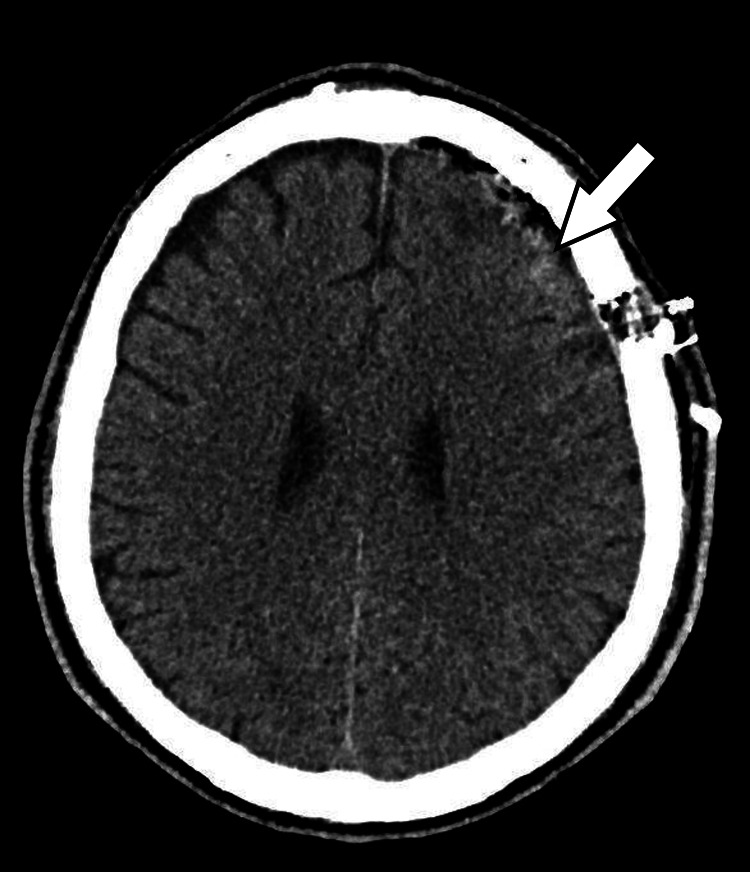
Axial view showing no residual left frontal lesion, with minimal hemorrhage at the surgical bed in keeping with post-operative changes

**Figure 6:**
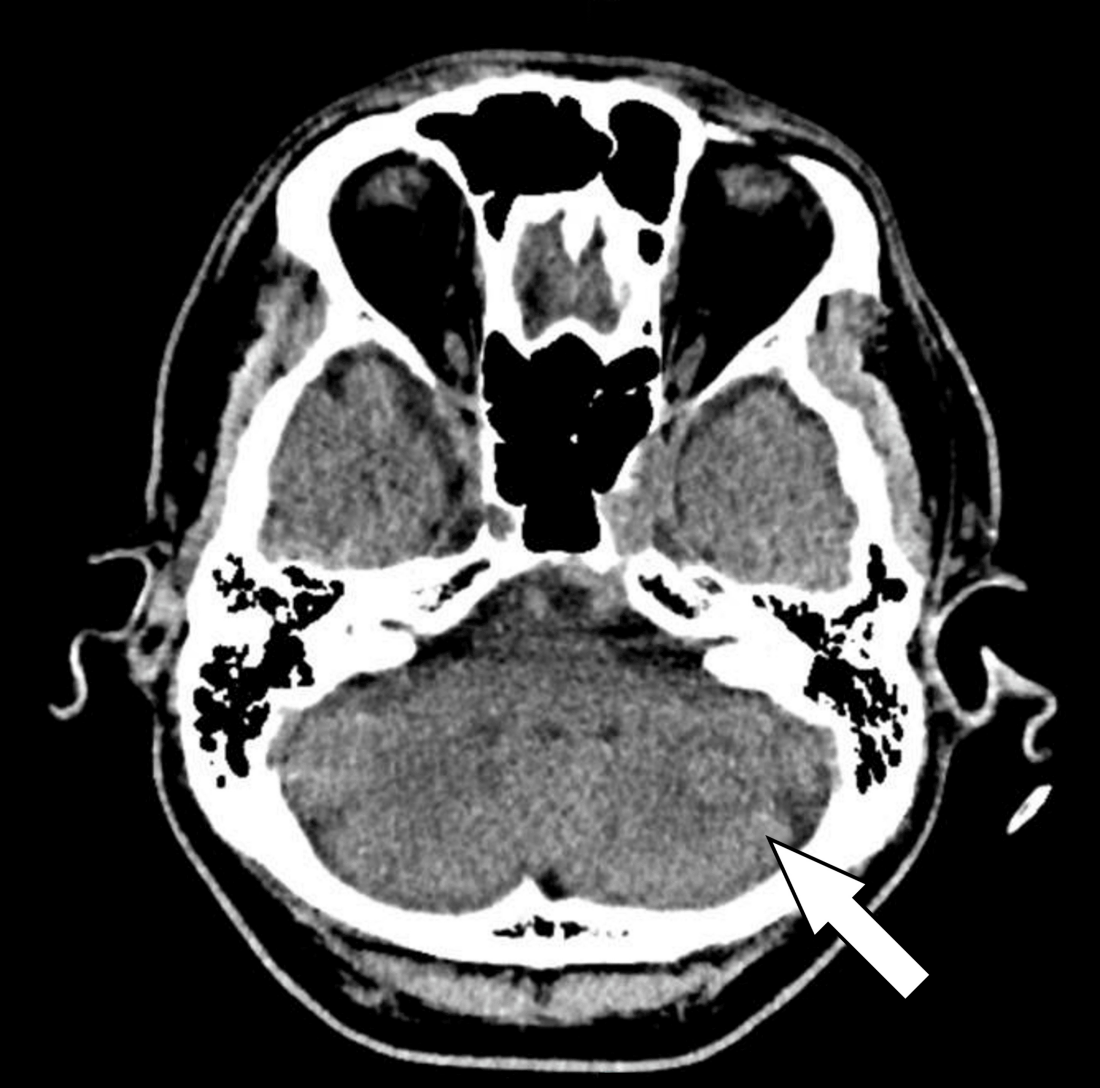
Axial view showing a left cerebellar lesion smaller in size compared to that in previous MRI brain

Initially, he was empirically treated as cerebral toxoplasmosis with a differential diagnosis of lymphoma with central nervous system (CNS) involvement. After two weeks of anti-toxoplasmosis treatment, the repeated CECT brain showed no response to the treatment. Thus, the patient was further evaluated with biopsy of the right temporalis muscle. The biopsy result of the right temporalis muscles revealed a benign fibroma with negative TB and fungal tissue cultures. Then, he was scheduled for bilateral tonsillectomy, and histopathology of the right tonsil revealed an EBV-SMT. He also underwent left frontal craniotomy with tumor debulking, and the histopathology revealed an EBV-SMT with negative non-tuberculous bacterium and mycobacterium PCR including negative tissue cultures for bacterial, TB, and fungal infection. His immediate post-operative CT brain plain revealed no apparent residual lesion in the frontal lobe with minimal intracranial hemorrhages at the surgical bed in keeping with post-operative changes and reducing the size of cerebellar lesions with no new lesions in the brain parenchyma (Figures 5, 6). He remains well with no permanent neurological deficits except for residual left cranial nerve palsy. Subsequently, he was started with ART (tenofovir- emtricitabine-efavirenz) after opportunistic infections were ruled out. The patient was discharged and followed up as an outpatient. After one month of operation, clinically he was well and his CD4 count and HIV viral load were 115 cells/uL and 73 copies/ml. He is planning for repeat MRI brain in another two months. His long-term plan for follow-up will be continuation of lifelong ART with periodic monitoring of HIV RNA viral load and clinical evaluation for any new signs and symptoms due to the tumor. In addition to this, oncology opinion was sought, there is no role for chemoradiotherapy and the mainstay of treatment is surgical resection and ART.

## Discussion

EBV-SMTs have been increasingly reported over the past few decades. These tumors are mainly categorized into three groups based on the underlying cause of immune deficiency, namely, HIV-related EBV-SMT, post-transplant-related EBV-SMT, and congenital immunodeficiency-related EBV-SMT [2]. In general, it is estimated that each of the above groups has <1-5% prevalence of EBV-SMTs [7]. The pathogenesis of EBV-SMTs remains largely speculative. The role of the immune system is however undisputable in the pathogenesis of these tumors. Specifically, the role of T cell and NK cell immunity in the prevention of these tumors is evident with an immunocompromised host being a condition sine qua non for the occurrence of these tumors [7]. It is also hypothesized that the mTOR/Akt pathway is involved in their pathogenesis, which is supported by reports of tumors responding to treatment with sirolimus in patients with post-transplant EBV-SMT. The clinical features of these tumors are not specific and mainly depend on the location, size of the tumors, and symptoms caused by compression or disruption of neighboring structures [7]. Most of these tumors involve the liver, but they can involve virtually any area of the body including the brain, spinal cord, lungs, gastrointestinal tract, adrenals, and bones [7]. HIV/AIDS patients have a predilection for CNS involvement including cerebral lobes, basal ganglia, cerebral sinuses, cerebellum, and intraspinal regions from cervical to sacrum. Among them, particularly common areas include parasellar regions like cavernous and sphenoid areas, which could be explained by the increased vascularity in these areas as it is hypothesized that these tumors arise from vascular smooth muscle cells [8]. While CNS is a common site of involvement, tonsils remain an extremely rare site to get involved, with only three cases of tonsillar EBV-SMTs reported worldwide [9,10]. Interestingly, these multifocal lesions are not considered metastasis, but rather independent tumors arising as a result of multiple infection events as evidenced by different clonality of viruses identified from different tumors in the same patient [5]. The diagnosis of these tumors can be elusive as they are quite rare entities. However, with an increased number of cases being reported, and as physicians and pathologists become more aware of these tumors, more cases are expected to be reported in the future. The gold standard of diagnosis remains biopsy-based histopathology with immunohistochemistry and EBV-encoded small RNA (EBER) in situ hybridization (ISH). Since there is no pathognomonic finding in radiology, a definitive diagnosis cannot be established without histopathology. CSF EBV DNA PCR has a role as a less invasive method of diagnostic workup for CNS lymphoma. However, there are no studies regarding this in the setting of CNS EBV-SMTs. Hence, the significance of positive CSF EBV DNA PCR in our patient for the diagnosis of EBV-SMTs with CNS involvement needs to be validated with further studies. When smooth muscle tumors appear in unusual sites or when immunodeficient patients present with multifocal tumors, it is pertinent to conduct further histological studies such as EBER ISH as only immunohistochemistry may lead to false negative results. Furthermore, we must not forget to do a thorough workup to look for other possibilities of opportunistic infections for immunocompromised status if a patient without a previously known immunodeficiency is diagnosed with an EBV-SMT. Another differential diagnosis that may be considered in the setting of HIV patients with a spindle cell tumor, include Kaposi sarcoma, mycobacterial spindle cell pseudotumor, and myopericytoma [11,12]. Given that EBV-SMTs are extremely uncommon tumors, it is not surprising that the treatment is not well established. The principle of treating these tumors centers around re-establishing host immunity. In patients with HIV/AIDS, this would include appropriate ART while in post-organ transplant patients this would entail a reduction of immunosuppressive therapy, with certain studies suggesting that switching of immunosuppressive therapy to mTOR inhibitors such as sirolimus improves outcome. In the case of congenital immunodeficiency syndromes, curative treatment of underlying immunodeficiency with allogeneic hematopoietic stem cell transplant seems to be an appropriate treatment for EBV-SMTs [1,7]. A lot of published studies focus on surgical resection for diagnosis as well as treatment. However, like our patient, since more than half of these patients present with multifocal tumors at the time of presentation, surgical resection may not always be feasible. In short, surgery should be reserved for diagnostic requirements, solitary lesions, and focal mass effect, and it may offer a survival benefit if the tumor is symptomatic and has intracranial invasion [8]. Radiotherapy and chemotherapy are other options that may be considered but do not show much overall benefit and cause more harm in most cases. Coming to the outcome and prognosis of these patients, generally, EBV-SMTs tend to be indolent, and locally invasive and rarely are the cause of death in these patients [13,14]. Usually, patients succumb to infectious complications such as opportunistic infections rather than tumor-related causes [1]. There is no demonstrable correlation between tumor multiplicity, histologic features, virulence mutations in the virus, and outcome from current studies [5,7,8]. Furthermore, factors such as age, gender, CD4 cell count, tumor type, tumor size, and location of the tumor had a noticeable impact on the outcome in HIV-related EBV-SMTs [12]. Prognostic data are mostly derived from a retrospective analysis of case series and overall survival is about 50% in HIV-related and post-transplant-related EBV-SMTs, whereas it is 0% in case of congenital immunodeficiency syndromes [13].

## Conclusions

EBV-SMTs even though rare should be considered in the differential diagnosis of mesenchymal tumors arising in immunocompromised patients, as well as smooth muscle tumors occurring in unusual sites. Even though it may be alarming to find multiple sites of involvement at the time of diagnosis, it is not cause for despair as they are not metastasis, but rather multiple events of infections. The mainstay of treatment is treatment of underlying immunodeficiency and surgical resection where feasible. Overall results are a good outcome. 

In the future, it may be beneficial to establish a clear treatment workup protocol for EBV-SMTs, particularly in HIV-infected patients. This would standardize care and potentially improve outcomes by ensuring timely diagnosis and management strategies, ultimately enhancing patient care.

## References

[1] Dekate J, Chetty R (2016). Epstein-Barr virus-associated smooth muscle tumor. Arch Pathol Lab Med.

[2] Chong YB, Lu PL, Ma YC, Yin HL, Chang CH (2022). Epstein-Barr virus-associated smooth muscle tumor and its correlation with CD4 levels in a patient with HIV infection. Front Cell Infect Microbiol.

[3] McClain KL, Leach CT, Jenson HB (1995). Association of Epstein-Barr virus with leiomyosarcomas in young people with AIDS. N Engl J Med.

[4] Lee ES, Locker J, Nalesnik M (1995). The association of Epstein-Barr virus with smooth-muscle tumors occurring after organ transplantation. N Engl J Med.

[5] Deyrup AT, Lee VK, Hill CE (2006). Epstein-Barr virus-associated smooth muscle tumors are distinctive mesenchymal tumors reflecting multiple infection events: a clinicopathologic and molecular analysis of 29 tumors from 19 patients. Am J Surg Pathol.

[6] Pitjadi TM, Grayson W (2019). Epstein-Barr virus-associated smooth muscle tumour: a case series with a significant proportion of tumours showing proclivity for cutaneous soft tissues. Dermatopathology (Basel).

[7] Magg T, Schober T, Walz C, Ley-Zaporozhan J, Facchetti F, Klein C, Hauck F (2018). Epstein-Barr Virus+ smooth muscle tumors as manifestation of primary immunodeficiency disorders. Front Immunol.

[8] Lau KW, Hsu YW, Lin YT, Chen KT (2021). Role of surgery in treating Epstein-Barr virus-associated smooth muscle tumor (EBV-SMT) with central nervous system invasion: a systemic review from 1997 to 2019. Cancer Med.

[9] Suwansirikul S, Sukpan K, Sittitrai P, Suwiwat S, Khunamornpong S (2012). Epstein-Barr virus-associated smooth muscle tumor of the tonsil. Auris Nasus Larynx.

[10] Dominelli GS, Jen R, Park K, Shaipanich T (2014). Tracheal Epstein-Barr virus-associated smooth muscle tumour in an HIV-positive patient. Can Respir J.

[11] Wang Y, Yang J, Wen Y (2022). Lessons from Epstein-Barr virus DNA detection in cerebrospinal fluid as a diagnostic tool for EBV-induced central nervous system dysfunction among HIV-positive patients. Biomed Pharmacother.

[12] Purgina B, Rao UN, Miettinen M, Pantanowitz L (2011). AIDS-related EBV-associated smooth muscle tumors: a review of 64 published cases. Patholog Res Int.

[13] Hussein K, Rath B, Ludewig B, Kreipe H, Jonigk D (2014). Clinico-pathological characteristics of different types of immunodeficiency-associated smooth muscle tumours. Eur J Cancer.

[14] Cela I, Shah NB, Bradly D, Loew J, Leslie W (2010). An Epstein-Barr virus-associated smooth muscle tumor successfully treated with surgical resection: a case report and literature review. Clin Adv Hematol Oncol.

